# Viral and murine interleukin-10 are correctly processed and retain their biological activity when produced in tobacco

**DOI:** 10.1186/1472-6750-9-22

**Published:** 2009-03-19

**Authors:** Luisa Bortesi, Marzia Rossato, Flora Schuster, Nicole Raven, Johannes Stadlmann, Linda Avesani, Alberto Falorni, Flavia Bazzoni, Ralph Bock, Stefan Schillberg, Mario Pezzotti

**Affiliations:** 1 Scientific and Technologic Department, University of Verona, Strada Le Grazie 15, 37134 Verona, Italy; 2 Department of Pathology, Section of General Pathology, University of Verona, Strada Le Grazie 8, 37134 Verona, Italy; 3 Institute for Molecular Biotechnology, Biology VII, RWTH, Worringerweg 1, 52074 Aachen, Germany; 4 Fraunhofer Institute for Molecular Biology and Applied Ecology (IME), Forckenbeckstrasse 6, 52074 Aachen, Germany; 5 Department for Chemistry, Glycobiology Division, University of Natural Resources and Applied Life Sciences, Muthgasse 18, 1190 Vienna, Austria; 6 Department of Internal Medicine, University of Perugia, Via E. Dal Pozzo, 06126 Perugia, Italy; 7 Max-Planck-Institute of Molecular Plant Physiology, Am Mühlenberg 1, 14476 Potsdam-Golm, Germany; 8 Department for Sciences, Technologies and Markets of Grapevine and Wine, University of Verona, Via della Pieve 70, 37029 San Floriano di Valpolicella (VR), Italy

## Abstract

**Background:**

Interleukin-10 (IL-10) is a potent anti-inflammatory cytokine, with therapeutic applications in several autoimmune and inflammatory diseases. Oral administration of this cytokine alone, or in combination with disease-associated autoantigens could confer protection form the onset of a specific autoimmune disease through the induction of oral tolerance. Transgenic plants are attractive systems for production of therapeutic proteins because of the ability to do large scale-up at low cost, and the low maintenance requirements. They are highly amenable to oral administration and could become effective delivery systems without extensive protein purification. We investigated the ability of tobacco plants to produce high levels of biologically-active viral and murine IL-10.

**Results:**

Three different subcellular targeting strategies were assessed in transient expression experiments, and stable transgenic tobacco plants were generated with the constructs that yielded the highest accumulation levels by targeting the recombinant proteins to the endoplasmic reticulum. The best yields using this strategy in T_1 _plants were 10.8 and 37.0 μg/g fresh leaf weight for viral and murine IL-10, respectively. The recombinant proteins were purified from transgenic leaf material and characterized in terms of their *N*-glycan composition, dimerization and biological activity in *in vitro *assays. Both molecules formed stable dimers, were able to activate the IL-10 signaling pathway and to induce specific anti-inflammatory responses in mouse J774 macrophage cells.

**Conclusion:**

Tobacco plants are able to correctly process viral and murine IL-10 into biologically active dimers, therefore representing a suitable platform for the production for these cytokines. The accumulation levels obtained are high enough to allow delivery of an immunologically relevant dose of IL-10 in a reasonable amount of leaf material, without extensive purification. This study paves the way to performing feeding studies in mouse models of autoimmune diseases, that will allow the evaluation the immunomodulatory properties and effectiveness of the viral IL-10 in inducing oral tolerance compared to the murine protein.

## Background

The production of biopharmaceuticals in transgenic plants has become more and more attractive over the last few years, primarily reflecting the low start-up and maintenance costs compared to fermenter-based production platforms and the ability to scale up production rapidly according to demand [1]. Plants can also be used as delivery systems for certain products, making extensive purification unnecessary. This applies in particular to products used for mucosal immunomodulation (e.g. oral vaccination or oral tolerance). Indeed, plant tissues are highly suitable for oral administration, allowing the direct delivery of recombinant protein drugs to the gut-associated lymphoid tissue (GALT) while encapsulating the proteins and protecting them from digestion [2,3].

With these advantages in mind, we investigated the possibility of using plants to produce the anti-inflammatory cytokine interleukin-10 (IL-10), a multifunctional cytokine from the alpha-helical bundle superfamily with diverse effects on most hemopoietic cell types [4]. While it plays a complex role in the immune system, the major activities of IL-10 are to inhibit cytokine production by macrophages and to suppress their accessory functions during T-cell activation [5,6]. Since this causes the termination of inflammatory responses, IL-10 is widely considered as an immunosuppressive and anti-inflammatory cytokine, and many investigations of IL-10 expression *in vitro*, in animal models and in human patients have indicated a significant role in inflammatory, malignant and autoimmune diseases, highlighting the potential clinical value of this cytokine [7]. Human IL-10 has been produced in stably transformed tobacco plants [8] and the ability of plant-produced human IL-10 to induce anti-inflammatory responses has also been demonstrated [9]. However, mammalian IL-10, including the human one, also presents several immunostimulatory properties (e.g., activation of dendritic cells, NK cells, and some T cells) which appear not to be negligible when IL-10 is used *in vivo *to induce tolerance [10].

Interestingly, IL-10 has orthologs in several virus genomes, and the IL-10 produced by Epstein-Barr virus (vIL-10) is particularly closely related to its human counterpart (71% and 84% identity at the nucleotide and amino acid sequence levels, respectively) and binds to both human and murine receptors [11]. Despite the sequence similarity, vIL-10 exhibits primarily the immune-inhibitory properties associated with the cellular cytokine (e.g., suppression of Th1-polarized responses and monocyte inhibition) but lacks many of the immunostimulatory properties associated with the human IL-10 (hIL-10) and murine IL-10 (mIL-10) [12]. This exacerbated inhibitory effect of vIL-10 makes it an even more attractive therapeutic candidate for tolerance induction, as the viral cytokine could have a profoundly different effect on the outcome of an *in viv*o immune response compared to hIL-10/mIL-10. [11].

One promising immunomodulatory application of IL-10 is to enhance the induction of oral tolerance to co-administered auto-antigens, leading to the prevention or treatment of autoimmune diseases with lower doses of tolerizing protein. We are particularly interested in the induction of oral tolerance for the prevention of type 1 diabetes mellitus (T1DM) which could be achieved by repeated oral administration of small doses of one of the major auto-antigens associated with the disease, the 65-kDa isoform of the enzyme glutamic acid decarboxylase (GAD65). We have already reported the characterization of transgenic tobacco plants producing immunoreactive GAD65 [13], although feeding studies in a mouse model have not yet been possible due to the low protein expression levels, requiring the consumption of unrealistically large amounts of plant material in order to achieve an oral tolerizing effect. However, simultaneous feeding with plant material containing IL-10 could reduce the amount of GAD65 necessary to induce oral tolerance. Additionally, IL-10 induces the formation of T regulatory-1 cells [10], and this should also drive the immune response towards active rather than a passive tolerance, which should in turn provide a more robust and long-lasting protection from the onset of the autoimmune disease. A comparative study, feeding either recombinant vIL-10 or the recombinant *endogenous *mIL-10 together with the GAD65 auto-antigen to NOD mice, would therefore allow to evaluate and assess the outcome of the different immunomodulatory properties of the viral cytokine in inducing oral tolerance in the animal model, and to evaluate the possibility of exploiting vIL-10 for the prevention of T1DM.

Here we describe for the first time the production of vIL-10 and mIL-10 in plants. Three different targeting strategies were used to identify the best subcellular compartment for IL-10 accumulation in tobacco leaves. Characterization of the plant-derived cytokines confirmed that tobacco plants can accumulate correctly-processed, dimeric IL-10 and that the recombinant protein retains the activity of its native counterpart. The impact of this achievement on the oral delivery of auto-antigens produced in plant tissues is discussed.

## Results

### Investigation of different targeting strategies in transient expression assays

**Figure 1:**
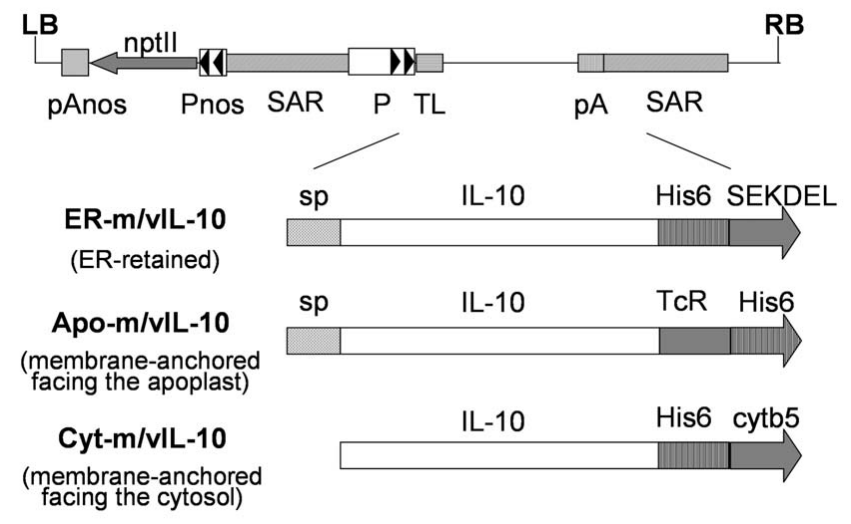
**T-DNA region of the pTRAk plant expression vector and gene cassettes targeting murine and viral IL-10 to different subcellular locations**. LB and RB, left and right border of the T-DNA; Pnos and pAnos, promoter and terminator of the nopaline synthase gene; nptII, coding sequence of the neomycin phosphotransferase gene; SAR, scaffold attachment region; P and pA, 35S promoter with duplicated enhancer and terminator of the cauliflower mosaic virus (CaMV) 35S gene; TL, 5' UTR of the tobacco etch virus (TEV); sp, native signal peptide; IL-10, mature IL-10 sequence; His_6_, histidine tag; SEKDEL, ER retention signal; TcR, C-terminal hinge and transmembrane domain of the human T-cell receptor; cytb5, transmembrane domain of the rat cytochrome-b5. Not drawn to scale.

**Figure 2:**
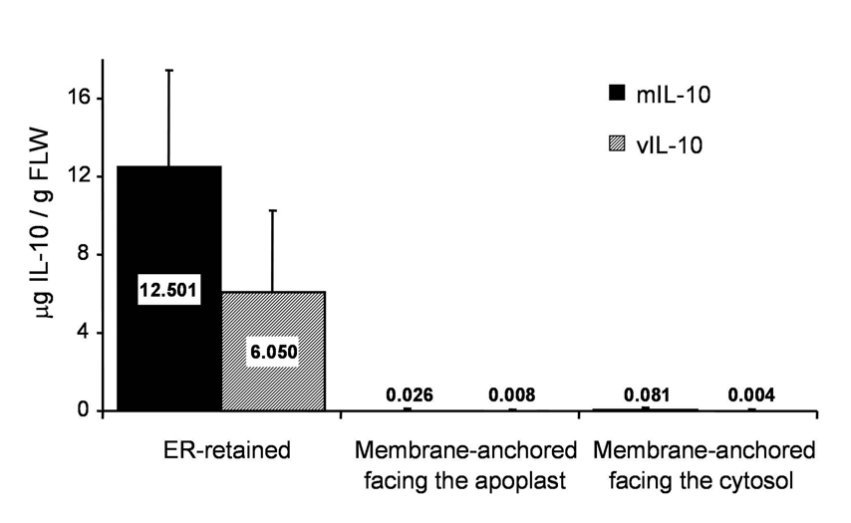
**Comparison of IL-10 levels in leaves agroinfiltrated with the different targeting constructs**. *N. tabacum *cv. Petit Havana SR1 leaves were vacuum-infiltrated with *A. tumefaciens *carrying the different plant expression constructs. For each construct, three to four leaves from different wild-type plants were infiltrated; they were incubated on wet filter paper for 3 d, with a 16-h photoperiod at ~20°C. Quantification of IL-10 accumulation levels in the leaf extracts was performed by sandwich ELISA. The gray and solid bars represent the data for the viral and murine IL-10, respectively; the localizations reported on the x axis refer to the constructs described in Figure 1. Values are the means ± SD of three independent experiments.

Three constructs each were designed for the mIL-10 and vIL-10 transgenes, targeting the recombinant proteins to three different sub-cellular localizations: (i) the endoplasmic reticulum (ER), achieved by retaining the native signal peptide and adding a C-terminal SEKDEL motif for ER retention (Fig. 1, ER-m/vIL-10) [14]; (ii) the plasma membrane (IL-10 facing the apoplast), achieved using the native signal peptide and adding the C-terminal hinge and transmembrane domain from the human T-cell receptor (Fig. 1, Apo-m/vIL-10) [15]; and (iii) the ER membrane (IL-10 facing the cytosol), achieved by deleting the native signal sequence and adding the C-terminal cytochrome b5 (cytb5) membrane anchor domain (Figure 1, Cyt-m/vIL-10) [14,16,17]. These constructs, all bearing a His_6_-tag at the C-terminus to facilitate protein purification, were generated by PCR and inserted into the plant expression vector pTRAk [18] placing the transgene under the control of the CaMV 35S promoter with duplicated enhancer (Figure 1). All plasmids were transferred into *Agrobacterium tumefaciens*, and transient expression in *Nicotiana tabacum *cv. Petit Havana SR1 leaves was carried out at least three times for each construct by agroinfiltration. Analysis of IL-10 accumulation in leaves using a sandwich enzyme-linked immunosorbent assay (ELISA; Figure 2), showed that both mIL-10 and vIL-10 accumulated to the highest levels in the ER (up to 10–16 μg/g fresh leaf weight (FLW)). Furthermore, immunoblot analysis of leaf extracts indicated that the recombinant proteins were correctly processed, as the molecular weights were consistent with those of the mature native polypeptides (taking into account the C-terminal His_6 _and SEKDEL tags; data not shown).

### Stable transformation of tobacco with constructs for ER retention

Based on the transient expression data, stable nuclear transformation of *N. tabacum *cv. Petit Havana SR1 was carried out using the ER-m/vIL-10 constructs. For each construct, 40 transgenic plants were regenerated and the amount of IL-10 in the leaves of the hemizygous T_0 _plants was estimated by ELISA. For mIL-10, the best yield was 21.3 μg/g FLW, whereas the best yield of vIL-10 was 8.9 μg/g FLW, mirroring the results of the transient expression assays. Transformants were fertile and the measurements were repeated in T_1 _plants, resulting in accumulation levels up to 37.0 μg/g FLW for mIL-10 and 10.8 μg/g FLW for vIL-10. Transgenic plants expressing vIL-10 grew more slowly than wild type plants and were stunted to an extent that correlated with the amount of recombinant protein accumulating in the leaves (see Additional file 1). The plants expressing mIL-10 did not display any mutant phenotype.

### Plant-derived mIL-10 and vIL-10 are dimeric

**Figure 3:**
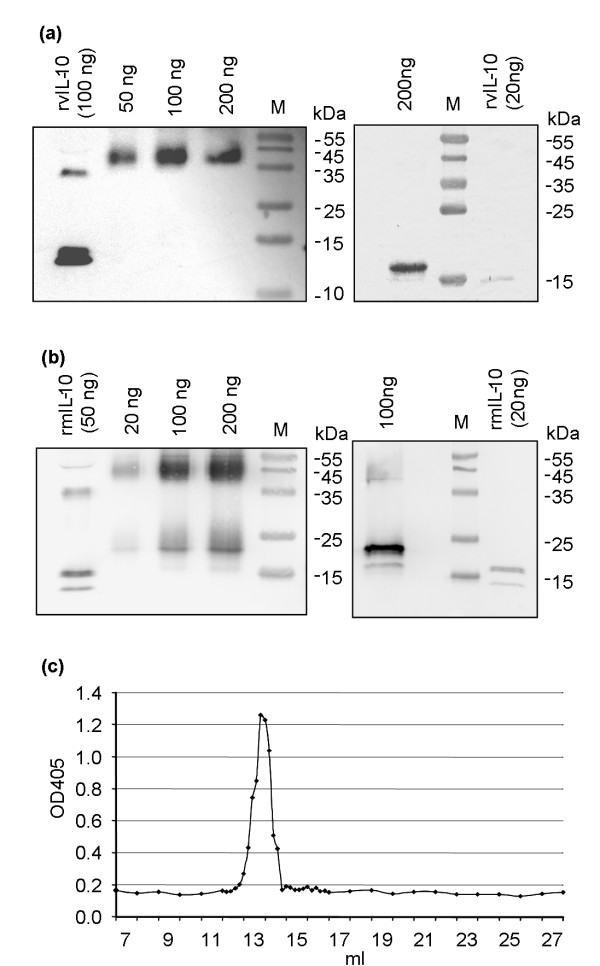
**Assessment of IL-10 integrity and dimerization**. Viral **(a) **and murine **(b) **IL-10, purified from transgenic tobacco leaf extracts by IMAC, were analyzed by immunoblotting after separation by non-reducing (left panels) and reducing (right panels) SDS-PAGE. Commercial recombinant viral IL-10 produced in *E. coli *(rvIL-10) or murine IL-10 produced in insect cells (rmIL-10) were used as controls, and different amounts of plant-derived IL-10 were loaded, as indicated on top of each lane. **(c) **Elution profile of plant-derived mIL-10 from a Superdex 200 column showing a single peak which, compared to the elution volumes of standard reference molecules on the same columns (not shown), matches with the expected size of the dimer. Detection of IL-10 in the elution fractions was carried out by ELISA.

The biological activity of IL-10 depends strictly on its dimeric state [19]. In order to determine whether or not mIL-10 and vIL-10 were properly assembled into dimers, the His_6_-tagged proteins were purified from stable transgenic tobacco leaves via immobilized-metal affinity chromatography (IMAC) on a nickel-nitrilotriacetic acid (Ni-NTA) column, concentrated, and fractionated by sodium dodecylsulfate polyacrylamide gel electrophoresis (SDS-PAGE) under either reducing or non-reducing conditions. Immunoblot analysis confirmed the correct molecular weights of both viral and murine IL-10 (19 and 21 kDa, respectively; Figures 3a and 3b, right panels). When separated under non-reducing conditions, the plant-derived vIL-10 predominantly formed stable dimers (Figure 3a, left panel), whereas mIL-10 was partly dimeric and partly monomeric (Figure 3b, left panel). Since commercial mIL-10 also gives rise to a monomer band under these conditions, this is most likely an artifact of the SDS-PAGE method. This was confirmed by gel filtration analysis, which showed that the plant-derived mIL-10 eluted as a single peak with a profile corresponding to the size of the dimeric form (Figure 3c).

### Investigation of N-glycosylation and signal peptide cleavage

**Figure 4:**
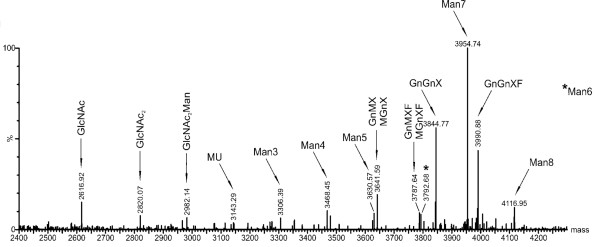
**Liquid chromatography-mass spectrometry (LC-MS) of the murine IL-10 glycosylated peptide EDNNCTHFPVGQSHMLLELR**. Murine IL-10 purified from stable transgenic leaf material was digested with trypsin and the peptides subjected to LC-MS analysis. The spectrum of the glycosylated peptide is shown. See  for an explanation of *N*-glycan abbreviations.

The mature mIL-10 polypeptide has two potential *N*-glycosylation acceptor sites, whereas vIL-10 has a single site. To determine the glycan composition, both plant-derived proteins were digested with trypsin and analyzed by mass spectrometry (MS). Despite the presence of a glycan acceptor site at Asn_127_, the plant-derived vIL-10 was not glycosylated. In contrast, mIL-10 was glycosylated, but only one of the two potential acceptor sites was used, as is the case for the native protein [20,21]. Unexpectedly for a protein bearing a SEKDEL tag, oligo-mannose-type (OMT) *N*-glycans accounted for only 46% of the total *N*-glycan population, with Man_7 _being the most abundant glycoform (Figure 4, *N*-glycan abbreviations according to Proglycan [22]). Complex type *N*-glycans accounted for 44% of the total, with GnGnXF and GnGnX being the most prominent. GnMX was present at lower levels, while the other complex type *N*-glycans were found in only trace amounts.

For both proteins, the identity of the N-terminal peptide was established by tandem MS analysis. The sequence of the mature mIL-10 polypeptide started with QYSRG while vIL-10 started with TDQCD, confirming co-translational ER translocation and correct cleavage of both signal peptides, as predicted by the SignalP 3.0 server [23].

### Biological activity of plant-derived mIL-10 and vIL-10

**Figure 5:**
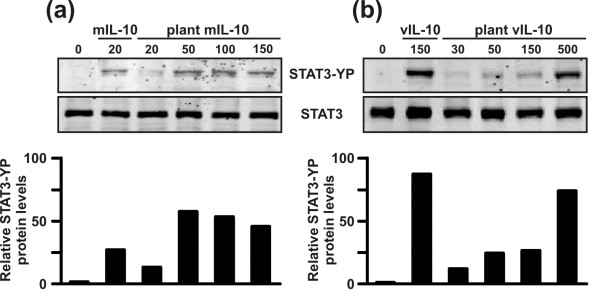
**Activation of STAT3 phosphorylation by murine and viral IL-10 produced in tobacco plants**. STAT3 phosphorylation (STAT3-YP) was assessed by immunoblot analysis on protein extracts of macrophage cells treated with increasing doses of plant-derived IL-10. **(a) **J774 cells were cultured for 15 min in the presence of eluate from wild type tobacco plants (lane 1; protein amount equivalent to the 150 ng/ml sample of the mIL-10-producing tobacco line), 20 ng/ml of commercial mIL-10 produced in insect cells (lane 2) or increasing concentrations of plant-derived mIL-10 (lanes 3–6); **(b) **J774 cells were cultured for 15 min in the presence of the eluate from purification of wild type tobacco plants (lane 1; protein amount equivalent to the 500 ng/ml sample of the viral IL-10-producing tobacco line), 150 ng/ml of commercial vIL-10 (lane 2) or increasing concentration of plant vIL-10 (lanes 3–6). Total cell extracts (50 μg) were separated by SDS-PAGE and immunoblots were performed as described in the Experimental Procedures section. One experiment representative of two is shown. The blots were scanned on the Odyssey Infrared Imaging System at 700 and 800 nm. The relative STAT3-YP levels, as quantified by the Odyssey software and normalized for the total STAT3, are reported below each panel.

All the anti-inflammatory effects of IL-10 require the activation of signal transducer and activator of transcription 3 (STAT3), which acts downstream of the IL-10 receptor in both mouse and human cellular models [11]. We therefore tested the ability of plant-derived mIL-10 and vIL-10 to phosphorylate the Tyr_705 _residue of STAT3. To exclude possible interference from plant proteins co-purifying with the recombinant IL-10 molecules, equal amounts of wild type leaf material were subjected to the same purification procedure on a Ni-NTA column, and the eluate was used as a control in all biological activity assays. Stimulation of the mouse macrophage cell line J774 with different doses of plant-derived mIL-10 and vIL-10 triggered STAT3 tyrosine phosphorylation within 15 min and in a dose-dependent manner (Figure 5). The concentration of plant-derived IL-10 required to elicit STAT3 tyrosine phosphorylation to a degree comparable to that observed in response to commercial mIL-10 (20 ng/ml) and vIL-10 (150 ng/ml) was 50 ng/ml for the murine protein (Figure 5a) and 500 ng/ml for the viral protein (Figure 5b). STAT3 phosphorylation resulted specifically from the presence of recombinant IL-10, since control eluate from wild type plant extracts had no detectable effect even at the highest dose tested (Figure 5).

**Figure 6:**
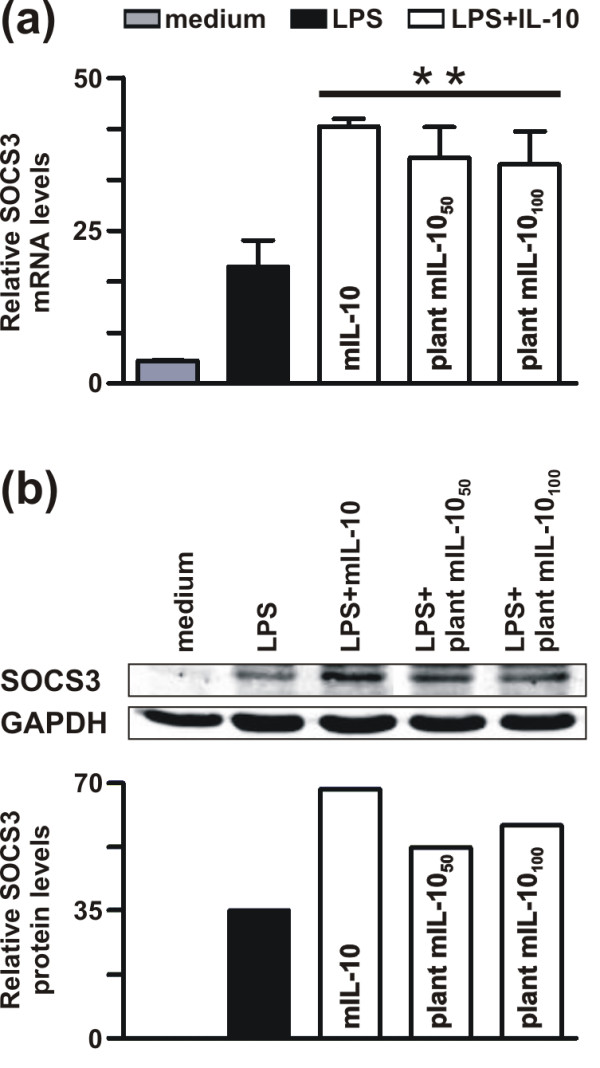
**Effect of plant-derived murine IL-10 on LPS-induced *SOCS3 *mRNA and SOCS3 protein expression**. **(a) **J774 cells were stimulated for 2 h with 100 ng/ml of LPS alone or in combination with commercial mIL-10 (20 ng/ml), or plant mIL-10 (50 and 100 ng/ml). Non stimulated cells ('medium') were included in the analysis to determine *SOCS3 *basal expression levels. Total RNA was extracted and then analyzed for *SOCS3 *mRNA expression by real time RT-PCR. The graph shows the *SOCS3 *mRNA levels (mean ± SD) assayed in triplicate and normalized to *GAPDH *expression. **(b) **J774 cells were incubated for 18 h in the presence of LPS alone (lane 2) or in combination with 20 ng/ml commercial IL-10 (lane 3) or 50 ng/ml (lane 4) or 100 ng/ml (lane 5) plant derived mIL-10. Non stimulated cells ('medium') were included in the analysis to determine SOCS3 basal expression levels. Whole cell extracts (50 μg) were immunoblotted using anti-NH_2 _terminus SOCS3 antibody (*upper panel*) and antibodies specific for GAPDH (*lower panel*), followed by incubation with AlexaFluor 680 goat anti-rabbit and IRDye 800 goat anti-mouse antibody. The relative levels of SOCS3 protein, as quantified by the Odyssey software and normalized for the total GAPDH content, are reported at the bottom of each lane. The data shown in (a) and (b) are representative of two independent experiments. ** p < 0.001.

**Figure 7:**
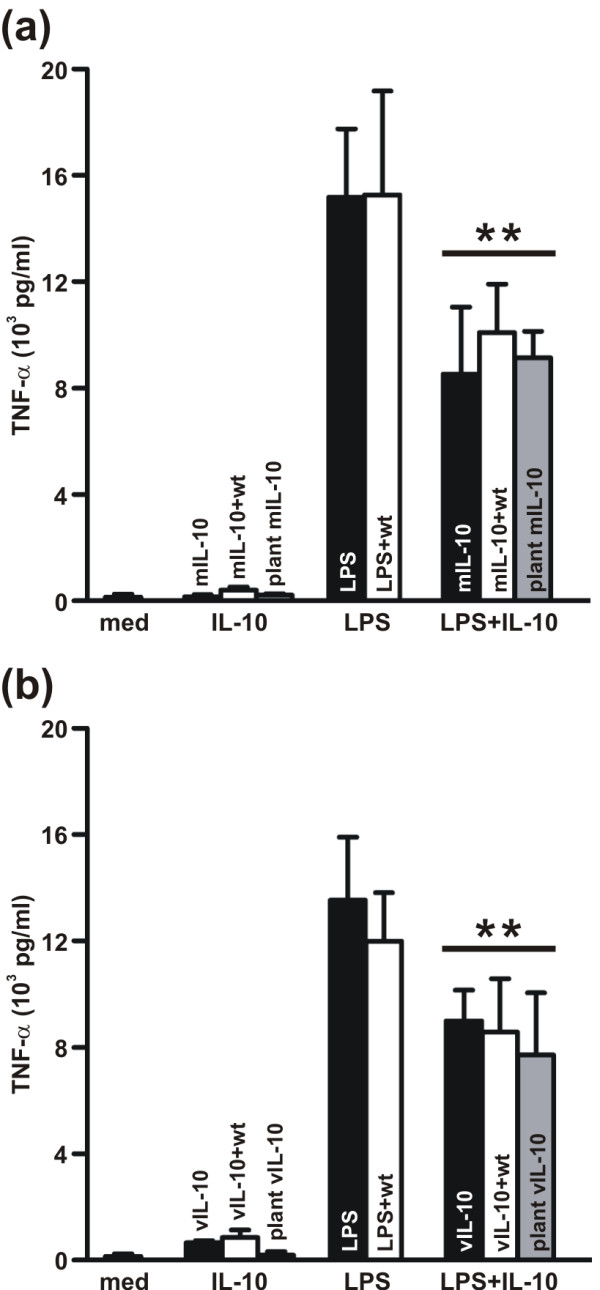
**Inhibition of LPS-induced TNFα production by plant-derived murine and viral IL-10**. J774 cells were stimulated for 18 h with IL-10 alone, LPS alone, or LPS plus IL-10. The eluate of the purification of wild type leaves (wt) was also included, alone or in combination with commercial IL-10, to exclude the interference of plant proteins with the assay. TNFα secretion in the culture medium (med) was quantified by ELISA and is reported as pg/ml supernatant. Values are the means ± SD of four independent experiments for the murine IL-10 (a) and three independent experiments for the viral IL-10 (b). ** p < 0.001.

It has been reported that suppressor of cytokine signaling 3 (SOCS3) is one of the targets of murine IL-10 [24,25]. Indeed, *SOCS3 *mRNA and SOCS3 protein expression in lipopolysaccharide (LPS)-stimulated J774 cells is enhanced in the presence of commercial mIL-10 [26]. Similarly, purified plant-derived mIL-10 increased the expression of LPS-induced *SOCS3 *mRNA (Figure 6a) and SOCS3 protein (Figure 6b). Finally, we verified that the plant-derived mIL-10 and vIL-10 were fully functional by testing their inhibitory activity on the LPS-induced production of tumor necrosis factor alpha (TNFα). The amount of TNFα released into the culture supernatant of J774 cells stimulated with LPS for 18 h was significantly reduced in the presence of plant-derived mIL-10 to the same extent observed with the commercial mIL-10 (Figure 7a). Similarly, the ability of plant derived vIL-10 to inhibit LPS-induced TNFα production was equivalent to that of its commercial counterpart (Figure 7b). Addition of control eluate from wild type plants had no effect on the J774 response to LPS, or the suppressive activity of IL-10 (Figure 7). These results confirmed that the inhibition of TNFα production was dependent on the recombinant cytokines, and not on any co-purified plant proteins.

## Discussion

We set out to generate transgenic tobacco plants producing biologically-active vIL-10 and mIL-10 at levels sufficient for raw transgenic leaf material to be used in animal feeding studies. Such studies would compare the two versions of IL-10 as potential adjuvants for the induction of oral tolerance to a co-fed auto-antigen in a mouse model of type-1 diabetes or other autoimmune diseases.

The intracellular targeting of a recombinant protein often has a significant effect on its yield and, in some cases, also on its biological properties. Therefore, we targeted both versions of IL-10 to three different subcellular compartments – the ER, cytosol and apoplast – in order to assess the impact on protein accumulation. Preliminary studies of murine IL-10 transient expression driven by a PVX-based system in *N. benthamiana *performed in our laboratory, had shown that the ER was by far the most suitable compartment for mIL-10 accumulation compared to the cytosol and the apoplast (ER-retained reached around 10 μg/g FLW; cytosolic and apoplastic expression was nearly undetectable as determined by immunoblot analysis; data not shown). As it has been reported that proteins associated with the plasma membrane are protected from degradation in the cytosol and the apoplast [15,27,28], in this work we used membrane anchors that allowed the recombinant proteins to be exposed to the apoplast or cytosol while retaining them in the membrane, in the attempt to increase the overall IL-10 accumulation levels. We evaluated the three targeting strategies by transient expression in tobacco leaves following agroinfiltration, since this provides a reliable indication of performance much more rapidly than the generation of stably transformed plants. Despite the addition of membrane anchors to stabilize the recombinant proteins in the cytosol and the apoplast, both versions of IL-10 accumulated to the highest levels in the ER, confirming our previous observations for mIL-10 and in line with what has been observed for hIL-10 stably expressed in tobacco [8]. Many other recombinant proteins have also accumulated to the highest levels in the ER when different targeting strategies were compared [29-31]. Our data therefore confirm the beneficial environment within the ER, favoring correct protein folding and assembly, and high protein stability.

In T_1 _transgenic plants, the highest level of mIL-10 accumulation was 37.0 μg/g FLW (0.6% total soluble protein (TSP)) and the highest level of vIL-10 accumulation was 10.8 μg/g FLW (0.1% TSP). These yields fall into the 0.1–1.0% TSP range typically observed for pharmaceutical proteins produced in nuclear transgenic plants [32] and are much higher than the 0.0055% TSP previously reported for hIL-10, which is very similar in structure to the murine and viral orthologs [8,33]. Surprisingly, the expression of vIL-10 caused a stunted phenotype whose severity correlated with the level of protein accumulation. Further investigation is required to clarify the direct impact of vIL-10 on plant development. The accumulation of both versions of IL-10 increased between the T_0 _and T_1 _generations, indicating that protein yields are partly dependent on the transgene copy number and/or zygosity. Further strategies could be employed to boost IL-10 accumulation, e.g. enhancing its stability through the use of fusion tags or through the co-expression of interacting molecules [34]. Nevertheless, the yields of both proteins are already sufficient for feeding studies in the mouse model. Indeed, on the basis of comparable experiments [35], 2–10 μg of IL-10 per day should be sufficient to elicit immunomodulatory effects and this would require the administration of a few milligrams of lyophilized plant tissue, which could be ingested by a mouse without substantially interfering with its dietary needs [9].

The expression of a recombinant protein containing a native signal peptide requires the signal peptide to be interpreted correctly by the heterologous host cell, otherwise the protein could be incorrectly cleaved or not cleaved at all. This is especially relevant for pharmaceutical proteins, where imprecise cleavage could affect the biological activity of the protein and might raise safety concerns [36]. The mIL-10 signal peptide was correctly processed in tobacco, as shown by sequencing the N-terminal peptide of the mature protein, which had the same QYSRE N-terminus previously reported for native mIL-10 [37]. There are at least two different N-terminal sequence reported for vIL-10: QCDNF and TDQCD. The former is claimed in several publications [38-40] and is most likely based on the prediction first reported by Moore and colleagues [20]. The latter is predicted by the latest version of the widely-used SignalP 3.0 Server [41,42], and is reported in at least one publication [43]. Tandem MS analysis of the N-terminal peptide of the purified vIL-10 produced in tobacco showed the N-terminal sequence to be TDQCD, in line with the prediction of the SignalP 3.0 Server. To our knowledge, this is the first time that the N-terminal amino acidic sequence of mature vIL-10 determined by bioinformatic tools, has been experimentally confirmed by the expression in a eukaryotic cell.

Although vIL-10 is not glycosylated [44], both the native and recombinant mIL-10 are heterogeneously *N*-glycosylated at an acceptor site near the N-terminus [20]. This glycosylation has no known influence on mIL-10 activity [11], but the presence of plant complex type *N*-glycans on therapeutic glycoproteins renders them potentially immunogenic and thus raises safety concerns, particularly in the case of parenteral administration. Even so the potential immunogenicity of plant glycans may also be important for orally administered proteins, especially in patients with severe food allergies. The glycan modifications that occur in the ER produce high-mannose-type *N*-glycans that are conserved between mammals and plants, so the use of a KDEL signal for ER retention not only increases protein yields, but also prevents the addition of plant complex type glycans. However, our *N*-glycan analysis indicated that mIL-10, although carrying a SEKDEL tag, is not efficiently retrieved to the ER, and is modified by enzymes located in the *trans*-Golgi network [45,46]. In addition, the presence of terminal GlcNAc residues on the complex glycan structures would suggest that mIL-10 escaping the ER is secreted by the plant cell and not, for example, directed to the vacuole [47]. The high efficiency of ER retrieval has been documented for a number of recombinant proteins produced in plants [48-50], but there are also several examples of KDEL-tagged recombinant proteins containing complex type glycans, indicating that the retrieval system can be leaky and must be evaluated on a case-by-case basis [51,52]. As immunoblot analysis of the purified protein confirmed that the KDEL-tag is still present (data not shown) and our protein yields are not high enough to suggest saturation of the KDEL retrieval machinery [53], the most likely explanation for the presence of complex type *N*-glycans on mIL-10 is that the tag is partially occluded following assembly of the IL-10 dimer, and is not completely accessible to the KDEL receptor. Localization studies will be performed in order to complement the *N*-glycan analysis, and to clarify where both versions of IL-10 precisely accumulate within the plant cell, particularly since vIL-10 cannot be traced using glycan structures.

Non-reducing SDS-PAGE followed by immunoblot analysis and (for mIL-10) gel filtration analysis confirmed that both vIL-10 and mIL-10 assemble into dimers in tobacco leaves. Moreover, it appears that the dimerization of both plant-derived versions of IL-10 is more efficient than that of their commercially produced counterparts. Since the higher dimer/monomer ratio is consistent regardless of the total amount of protein loaded onto the gel, we can rule out the possibility that this is an artifact caused by high protein concentration. It is also unlikely that the presence of plant glycans is responsible, since vIL-10 is not glycosylated. The most probable explanation is that the presence of the C-terminal SEKDEL and His_6 _tags may enhance the stability of the dimers. However, to confirm this assumption, we would need to express non-tagged versions of the proteins and compare the dimer/monomer ratios. Increased stability of plant-derived IL-10 dimers was also observed for hIL-10 by Menassa and colleagues [33]. Interestingly, hIL-10 was not glycosylated and the C-terminal tags had been cleaved from the plant-derived hIL-10 by thrombin digestion, which should remove all additional amino acids except a lysine and a valine.

The ability of both vIL-10 and mIL-10 to form dimers indicated that the recombinant proteins were likely to retain their biological activity, a hypothesis we tested by exposing LPS-stimulated J774 mouse macrophage cells to different amounts of recombinant IL-10 and assaying the effect on downstream components of the signaling pathway. We observed a clear dose-dependent effect on STAT3 phosphorylation after treatment with both varieties of IL-10, indicating that the plant-derived proteins properly interacted with IL-10 receptor and initiated the signal transduction cascade. Moreover, mIL-10 was able to upregulate the expression of the intracellular negative regulator of cytokine responses, SOCS3. Most importantly, given that IL-10 is best known for its ability to inhibit pro-inflammatory cytokine and chemokine expression in LPS-stimulated macrophages, our experiments demonstrated that both mIL-10 and vIL-10 are able to inhibit the LPS-induced production of TNFα. Higher doses of the plant-derived proteins as compared to the corresponding commercial IL-10 products were required, perhaps because of the presence of the C-terminal SEKDEL and His_6 _tags. Nevertheless, this will have no impact on the planning of feeding studies since the dose of IL-10 necessary to enhance oral tolerance needs to be determined empirically by administering different amounts of transgenic plant material to the mice.

## Conclusion

Taken together, these results clearly demonstrate that tobacco plants can express the viral and murine IL-10 genes, process and assemble the corresponding proteins into functional, biologically-active dimers. The accumulation levels of both viral and murine IL-10 in tobacco leaves are high enough to provide sufficient material for oral administration in oral tolerance studies using the available mouse models. This will allow to determine whether simultaneous feeding with plant material containing GAD65 and IL-10 could reduce the amount of auto-antigen necessary to prevent the onset of T1DM, and to comparatively evaluate the effectiveness of the viral and murine IL-10 as immunomodulators.

## Methods

### Plant transformation vectors

Six different constructs were generated using the polymerase chain reaction (PCR) to amplify the murine and viral IL-10 cDNA sequences from the pBluescriptSKII+mIL-10 [54] and pAAV-vIL-10 [55] plasmids, respectively, with forward primers incorporating an *Nco*I restriction site at the 5' end (for amplification of the complete IL-10 coding region including the native signal peptide), or a *Bsp*HI site (for amplification of the mature mIL-10 sequence) or an *Xmn*I site (for amplification of the mature vIL-10 sequence). The appropriate targeting and His_6_-tag sequences were added by PCR in-frame to the 3' end of the IL-10 coding region, and an *Xba*I site was created at the 3' end of all the constructs. The 3' nucleotide sequence for the ER-retained versions of IL-10 (ER-m/vIL-10) was 5 '-*CAT CAC CAT CAC CAT CAC *TCT GAG AAA GAT GAG CTC taa ACC GTC TAG AGC-3'. The sequence of the apoplast constructs (Apo-m/vIL-10) was 5'-GGT AGA GCA GAC TGT GGC TTT ACC TCG GTG TCC TAC CAG CAA GGG GTC CTG TCT GCC ACC ATC CTC TAT GAG ATC CTG CTA GGG AAG GCC ACC CTG TAT GCT GTG CTG GTC AGC GCC CTT GTG TTG ATG GCA ATG GTC AAG AGA AAG GAT TTC *CAT CAC CAT CAC CAT CAC *taa ACC GTC TAG AGC-3'. That of the cytosolic constructs (Cyt-m/vIL-10) was 5'-*CAT CAC CAT CAC CAT CAC *GTC GAG TCT AAT TCC AGT TGG TGG ACC AAC TGG GTG ATC CCA GCC ATC TCA GCC CTG GTG GTA GCT CTG ATG TAT CGC CTC TAC ATG GCA GAA GAT taa ACC GTC TAG AGC-3'. In each case, the nucleotides encoding for the His_6_-tag are in italic, the stop codons are shown in lower case, and the *Xba*I sites are underlined. The resulting amplification products were digested with the appropriate restriction enzymes and inserted into the pTRAk binary vector, a derivative of pPAM (GenBank: AY027531) [18]. The plasmids were electroporated into *A. tumefaciens *strain GV3101::pMP90RK [56].

### Transient and stable expression of IL-10 in tobacco

*Agrobacterium*-mediated transient expression (agroinfiltration) was carried out essentially as described by Kapila *et al*. [57]. The bacterial cultures were adjusted to OD_600 _= 1 with 2× induction medium (10% (w/v) sucrose, 3.6% (w/v) glucose, 8.6% (w/v) Murashige-Skoog salts) and water, and induced for an additional 2 h with 200 μM acetosyringone. *N. tabacum *cv. Petit Havana SR1 leaves were vacuum-infiltrated at 70 mbar for 20 min in the bacterial suspension then placed in plastic trays with moistened Whatman paper. The trays were sealed with Saran wrap and incubated at 25°C with a 16-h photoperiod, 7000 Lux, for 3 d.

Stable transgenic tobacco (*N. tabacum *cv. Petit Havana SR1) plants were produced using the leaf disc transformation method [58]. T_0 _plants were grown on Murashige-Skoog medium containing 100 mg/l kanamycin prior to transfer to soil in the glasshouse, and then selfed to produce the T_1 _generation. Transgenic T_1 _plants were also selected on kanamycin-containing medium and maintained in soil.

### Immunoblot analysis

Soluble proteins were extracted from leaf discs with three volumes of PBS (pH 7.4) containing 0.05% (w/v) Tween-20, 5 mM EDTA and 2% (w/v) polyvinylpolypyrrolidone (PVPP). Equal volumes of the supernatant were either separated by SDS-PAGE on a 17% (w/v) gel prior to blotting, or spotted directly onto a nitrocellulose membrane, blocked for 1 h with 5% (w/v) non-fat milk and incubated overnight at 4°C with either a rat anti-mouse IL-10 antibody (BD Pharmingen, San Diego, CA, USA) diluted 1:2000 or a rat anti-human IL-10 and viral IL-10 (BD Pharmingen) antibody diluted 1:1000. The membrane was then incubated for 1 h at room temperature with an alkaline phosphatase (AP)-conjugated rabbit anti-rat immunoglobulin G (IgG) (Sigma-Aldrich, St. Louis, Mo, USA) diluted 1:5000. The signal was detected by incubation with nitroblue tetrazolium and 5-bromo-4-chloro-3-indolyl phosphate (NBT-BCIP). Recombinant mIL-10 produced in insect cells (BD Pharmingen) and vIL-10 produced in *Escherichia coli *(R&D Systems, Minneapolis, MN, USA) were used as standards.

### Quantification of IL-10 by ELISA

Microtiter plates (High-binding; Greiner Bio-One GmbH, Frickenhausen, Germany) were coated overnight at 4°C with either a rat anti-mouse IL-10 antibody (BD Pharmingen) diluted 1:1000 in 0.2 M sodium phosphate, pH 6.5, or a rat anti-human IL-10 and viral IL-10 antibody (BD Pharmingen) diluted 1:2000 in 50 mM sodium carbonate buffer, pH 9.6. Wells were blocked with 1% (w/v) bovine serum albumin (BSA) in PBS containing 0.05% (w/v) Tween-20 (PBST) for at least 1 h. Samples diluted in PBS were applied to the coated plates and incubated overnight at 4°C. The corresponding biotinylated antibodies (554465 and 554499; BD Pharmingen) were added to the plates at 1:1000 dilution in PBST and incubated for 1 h at room temperature. AP-conjugated streptavidin (Jackson ImmunoResearch Laboratories, West Grove, PA, USA) was added to the wells at 1:2500 dilution in PBST, and incubated for 30 min at room temperature before the signal was detected with *p*-nitrophenyl phosphate. After 15–20 min, the signal was quantified by measuring the absorbance at 405 nm. Ten two-fold serial dilutions of the standards described in the previous section, starting from a concentration of 50 ng/ml for the murine and 1 ng/ml for the viral IL-10, respectively, were used as positive controls, and the linear range of the dose-response curve obtained was used for quantification of IL-10 in the samples. Extract from wild-type tobacco was used as negative controls. The plates were washed with PBST between incubations.

### Purification of IL-10 by IMAC

After removing the midrib, the leaf tissue was ground in a mortar with liquid nitrogen and extracted with three volumes of buffer (PBS, 10 mM ascorbic acid, 0.1% (w/v) Tween-20, pH 6.0). Insoluble material was removed by centrifugation (15 min, 15,000 × *g*, 4°C). The supernatant was passed through a paper filter (#MN615, Macherey-Nagel, Düren, Germany), adjusted to 500 mM NaCl, 5 mM imidazol, pH 8.0, and placed for 1 h on ice, gently shaking. After another centrifugation step (30 min, 30,000 × *g*, 4°C), the clear supernatant was loaded on a disposable column (Bio-Rad, Hercules, CA, USA) packed with 1 ml Ni-NTA Superflow resin (QIAGEN, Hilden, Germany) using a flow rate of approximately 2 ml/min. The column was extensively washed with PBS (pH 8.0) containing 500 mM NaCl and 5 mM imidazol to remove non-specifically bound material and the IL-10 was eluted with PBS containing 500 mM NaCl and 500 mM imidazol (pH 8.0). IL-10 fractions were pooled, dialyzed against water, and lyophilized. Lyophilized material was then dissolved in PBS (pH 7.4), the preparation was centrifuged to remove residual insoluble material and IL-10 concentration in the supernatant was determined by ELISA.

### Gel filtration

Gel filtration was performed using a Superdex 200 (10/30) column (GE Healthcare, Uppsala, Sweden), with PBS (pH 7.4) as the eluent and a flow rate of 0.35 ml/min. Purified recombinant mIL-10 (450 ng) was loaded on the column, and 1-ml fractions were collected for the elution volumes 7–12 ml and 17–28 ml. Finally, 0.2-ml fractions were collected from 12 to 17 ml. IL-10 was detected by ELISA.

### MS analysis of tryptic peptides and glycopeptides

Murine and viral IL-10 purified from leaf material by IMAC were separated on a 17% (w/v) gel by SDS-PAGE under reducing conditions, and the proteins were visualized by Coomassie brilliant blue staining. The bands were then excised, destained, carbamidomethylated, digested with trypsin and extracted from gel pieces, as previously described [59,60]. Subsequent fractionation of the peptides by capillary reversed-phase chromatography, with detection by a quadrupole time-of-flight (Q-TOF) Ultima Global mass spectrometer (Waters Micromass, Manchester, UK), was performed as described previously [59,61]. The MS data from the tryptic peptides were compared with data sets generated by *in silico *tryptic digestion of the recombinant protein sequences using the PeptideMass program [59,60,62]. Sequences of the N-terminal peptides were confirmed by tandem MS experiments. Data were analyzed with MassLynx 4.0 SP4 Software (Waters Micromass, Milford, MA, USA).

### Characterization of IL-10 biological activity

The functional activity of murine and viral IL-10 produced by tobacco plants was assessed in the mouse macrophage cell line J774 (provided by Dr. V. Kruys, Université Libre de Brussels, Belgium). Cells were resuspended in Dulbecco's modified Eagle's medium (DMEM) supplemented with 5% (w/v) low endotoxin fetal bovine serum (FBS) (both from BioWhittaker, Walkersville, MD, USA), seeded at a density of ~4 × 10^5^/ml in 48-well tissue culture plates and then stimulated with ultra-pure *E. coli *LPS (0111:B4 strain, InvivoGen, San Diego, CA, USA), in the presence or absence of recombinant murine IL-10 (20 ng/ml, BD Pharmingen), or recombinant viral IL-10 (300 ng/ml, R&D Systems), or the indicated doses of murine and viral IL-10 purified from tobacco plants (or corresponding negative control extracts from wild type leaves). The ability of IL-10 to activate STAT3 and to upregulate SOCS3 protein expression was evaluated by immunoblot analysis using cell lysates prepared as previously described [63]. Briefly, J774 extracts were prepared with lysis buffer (20 mM HEPES, 420 mM, NaCl, 1 mM EDTA, 1 mM EGTA, 1% (v/v) Nonidet-P40, 20% (v/v), glycerol, 1 mM DTT) containing inhibitors of proteases (5 μg/m leupeptin, 5 μg/ml pepstatin, 1 mM phenylmethylsulfonyl fluoride, 5 mg/ml α1-antitrypsin) and phosphatases (1 mM Na_3_VO_4_, 20 μM phenylarsine oxide, 50 mM NaF). We then separated 50 μg of J774 lysates by SDS-PAGE and transferred the proteins to a nitrocellulose membrane. For tyrosine-phosphorylated and total STAT3 detection, two-color immunoblots were performed with rabbit anti-phospho-STAT3 (Tyr705) and mouse anti-STAT3 124H6 antibodies (both from Cell Signaling, Denver, MA, USA), respectively, both diluted 1:1000. For SOCS3 and glyceraldehyde-3-phosphate dehydrogenase (GAPDH), two-color immunoblots were performed with rabbit anti-NH_2 _terminus SOCS3 (1:150 dilution; Immuno-Biological Laboratories, Tokio, Japan) and mouse anti-GAPDH (1:4000 dilution; Ambion, Austin, TX, USA) antibodies, respectively. Detection was simultaneously carried out with Alexa Fluor^R^680 goat anti-rabbit antibody (1:5000 dilution; Molecular Probes™, Invitrogen, Carlsbad, CA, USA) and IRDye™800-conjugated goat anti-mouse IgG (1:2500 dilution; Rockland, Gilbertsville, PA, USA) secondary antibodies. Blotted proteins were detected and quantified using the Odyssey infrared imaging system (LI-COR Biosciences, Lincoln, NE, USA) and software provided by the manufacturer.

*SOCS3 *gene expression was quantified by real-time RT-PCR. Briefly, total RNA was extracted from 1 × 10^6 ^J774 cells using the RNeasy mini kit (QIAGEN), according to the manufacturer's protocol. Reverse transcription was carried out using SuperScript II (Invitrogen), and real-time RT-PCR was performed in triplicate from 10 ng cDNA using the SYBR Green real-time master mix "SYBR Premix Ex Taq™" (Takara Bio Inc., Otsu, Shiga, Japan), in the presence of 200 nM specific primer pairs purchased from Invitrogen: SOCS3 forward 5'-CCTTTGTAGACTTCACGGCT-3', reverse 5'-TTTGGAGCTGAAGGTCTTGAG-3', GAPDH forward 5'-TGGCCTCCAAGGAGTAAGAA-3', reverse 5'-GGTCTGGGATGGAAATTGTG-3'. The reaction conditions performed by the DNA Engine Opticon 2 System (MJ Research, Waltham, MA, USA), were as follows: 95°C for 10 s, followed by 40 cycles of 95°C for 10 s and 60°C for 40 s. Data were calculated with LinReg PCR 7.0 and Q-Gene software  and then expressed as mean normalized expression (MNE) units after GAPDH normalization.

Determination of TNFα levels in cell-free supernatants was performed using a commercial ELISA kit (DY410; R&D Systems), according to the manufacturer's instructions.

### Statistical analysis

Statistical changes in TNFα production and *SOCS3 *mRNA expression levels were determined using the one-way ANOVA with α set to 0.05, according to the Newman-Keuls test.

## Authors' contributions

LB participated in the design of the study, generated the constructs, carried out the agroinfiltration experiments, analyzed the IL-10 accumulation levels, purified the recombinant proteins, carried out the STAT3 phosphorylation assay, the LPS induction assay and TNFα quantification and wrote the manuscript. MR carried out the RNA extraction and RT-PCR analysis, did the SOCS3 detection by Immunoblot, helped with the performance and did the evaluation of all the biological activity assays, participated in drafting the manuscript. FS carried out the plant stable transformation and helped with interpretation of the results. JS performed the *N*-glycan analysis and N-terminus sequence determination. NR and LA participated in experiment design and supervision, analysis and interpretation of the data, and in critical reading of the manuscript. AF participated in the conceiving and designing of the study and in critical reading of the manuscript. FB designed, supervised and interpreted the biological activity assays and helped writing the manuscript. RB, SS and MP conceived the study, participated in its design and coordination, interpretation of data and critical reading of the manuscript. All authors read and approved the final manuscript.

## Supplementary Material

Additional file 1 **Three independent primary transformants (8 weeks after transfer to soil) are shown and viral IL-10 accumulation levels, determined by ELISA, are indicated for each plant. **The correlation between the viral IL-10 accumulation levels and the stunted phenotype was observed both in the T_0 _and T_1 _generation plants. Click here for file
