# Supplementary figures and images for: Viral and murine interleukin-10 are correctly processed and retain their biological activity when produced in tobacco
Source: BMC Biotechnol. 2009 Mar 19;9:22. doi: 10.1186/1472-6750-9-22 (PMC2667500; doi:10.1186/1472-6750-9-22)

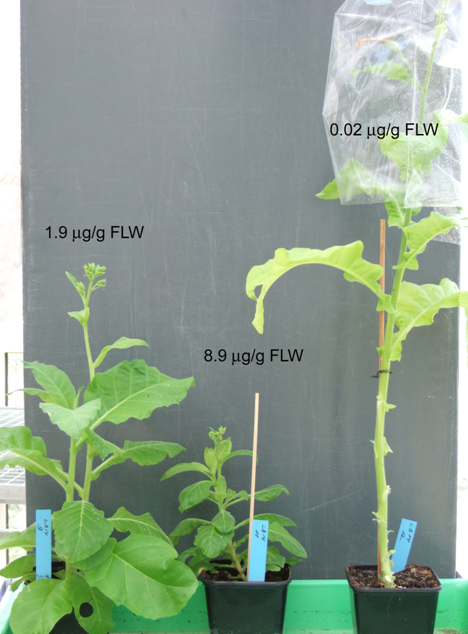

Supplement: Additional file 1 — Three independent primary transformants (8 weeks after transfer to soil) are shown and viral IL-10 accumulation levels, determined by ELISA, are indicated for each plant. The correlation between the viral IL-10 accumulation levels and the stunted phenotype was observed both in the T0 and T1 generation plants. [file 1472-6750-9-22-S1.tiff]
